# Quantifying hospital-associated costs, and accompanying travel costs and productivity losses, before and after withdrawing TNF-α inhibitors in juvenile idiopathic arthritis

**DOI:** 10.1093/rheumatology/kead688

**Published:** 2023-12-20

**Authors:** Anna A Florax, Martijn J H Doeleman, Sytze de Roock, Naomi van der Linden, Ellen Schatorjé, Gillian Currie, Deborah A Marshall, Maarten J IJzerman, Rae S M Yeung, Susanne M Benseler, Sebastiaan J Vastert, Nico M Wulffraat, Joost F Swart, Michelle M A Kip, for UCAN-CAN DU and UCAN CURE Consortia, Rae S M Yeung, Nico Wulffraat, Susanne M Benseler, Joost F Swart, Sebastiaan J Vastert, Marinka Twilt, Deborah A Marshall, Joseph Cafazzo, Rae S M Yeung, Susanne M Benseler, Deborah A Marshall, Joseph Cafazzo, Maarten J Ijzerman, Nico Wulffraat, Joost F Swart, Sebastiaan J Vastert, Marinka Twilt

**Affiliations:** Department of Health Technology and Services Research, Faculty of Behavioural, Management and Social Sciences, Technical Medical Centre, University of Twente, Enschede, The Netherlands; Department of Pediatric Rheumatology, Division of Paediatrics, University Medical Center Utrecht, Wilhelmina Children’s Hospital, Utrecht, The Netherlands; Faculty of Medicine, Utrecht University, Utrecht, The Netherlands; Department of Pediatric Rheumatology, Division of Paediatrics, University Medical Center Utrecht, Wilhelmina Children’s Hospital, Utrecht, The Netherlands; Faculty of Medicine, Utrecht University, Utrecht, The Netherlands; Department of Health Technology and Services Research, Faculty of Behavioural, Management and Social Sciences, Technical Medical Centre, University of Twente, Enschede, The Netherlands; Department of Paediatric Rheumatology, St Maartenskliniek, Nijmegen, The Netherlands; Department of Paediatric Rheumatology and Immunology, Amalia Children’s Hospital, Radboud University Medical Center, Nijmegen, the Netherlands; Department of Community Health Sciences, Cumming School of Medicine, University of Calgary, Calgary, AB, Canada; Department of Paediatrics, Cumming School of Medicine, University of Calgary, Calgary, AB, Canada; Alberta Children’s Hospital Research Institute, University of Calgary, Calgary, AB, Canada; Department of Medicine, Cumming School of Medicine, University of Calgary, Calgary, AB, Canada; Department of Community Health Sciences, Cumming School of Medicine, University of Calgary, Calgary, AB, Canada; Alberta Children’s Hospital Research Institute, University of Calgary, Calgary, AB, Canada; Department of Medicine, Cumming School of Medicine, University of Calgary, Calgary, AB, Canada; Department of Health Technology and Services Research, Faculty of Behavioural, Management and Social Sciences, Technical Medical Centre, University of Twente, Enschede, The Netherlands; Division of Rheumatology, The Hospital for Sick Children, Department of Paediatrics, Immunology and Institute of Medical Science, University of Toronto, Toronto, ON, Canada; Alberta Children’s Hospital Research Institute, University of Calgary, Calgary, AB, Canada; Division of Rheumatology, Department of Pediatrics, Alberta Children’s Hospital, Cumming School of Medicine, University of Calgary, Calgary, AB, Canada; Department of Pediatric Rheumatology, Division of Paediatrics, University Medical Center Utrecht, Wilhelmina Children’s Hospital, Utrecht, The Netherlands; Faculty of Medicine, Utrecht University, Utrecht, The Netherlands; European Reference Network RITA (Rare Immunodeficiency Autoinflammatory and Autoimmune Diseases Network); Department of Pediatric Rheumatology, Division of Paediatrics, University Medical Center Utrecht, Wilhelmina Children’s Hospital, Utrecht, The Netherlands; Faculty of Medicine, Utrecht University, Utrecht, The Netherlands; European Reference Network RITA (Rare Immunodeficiency Autoinflammatory and Autoimmune Diseases Network); Department of Pediatric Rheumatology, Division of Paediatrics, University Medical Center Utrecht, Wilhelmina Children’s Hospital, Utrecht, The Netherlands; Faculty of Medicine, Utrecht University, Utrecht, The Netherlands; European Reference Network RITA (Rare Immunodeficiency Autoinflammatory and Autoimmune Diseases Network); Department of Health Technology and Services Research, Faculty of Behavioural, Management and Social Sciences, Technical Medical Centre, University of Twente, Enschede, The Netherlands; Department of Pediatric Rheumatology, Division of Paediatrics, University Medical Center Utrecht, Wilhelmina Children’s Hospital, Utrecht, The Netherlands

**Keywords:** juvenile idiopathic arthritis, DMARD, biologics, TNFi, treatment, withdrawal, costs

## Abstract

**Objective:**

To quantify differences in hospital-associated costs, and accompanying travel costs and productivity losses, before and after withdrawing TNF-α inhibitors (TNFi) in JIA patients.

**Methods:**

This was a retrospective analysis of prospectively collected data from electronic medical records of paediatric JIA patients treated with TNFi, which were immediately discontinued, spaced (increased treatment interval) or tapered (reduced subsequent doses). Costs of hospital-associated resource use (consultations, medication, radiology procedures, laboratory testing, procedures under general anaesthesia, hospitalization) and associated travel costs and productivity losses were quantified during clinically inactive disease until TNFi withdrawal (pre-withdrawal period) and compared with costs during the first and second year after withdrawal initiation (first and second year post-withdrawal).

**Results:**

Fifty-six patients were included of whom 26 immediately discontinued TNFi, 30 spaced and zero tapered. Mean annual costs were €9165/patient on active treatment (pre-withdrawal) and decreased significantly to €5063/patient (−44.8%) and €6569/patient (−28.3%) in the first and second year post-withdrawal, respectively (*P* < 0.05). Of these total annual costs, travel costs plus productivity losses were €834/patient, €1180/patient, and €1320/patient in the three periods respectively. Medication comprised 80.7%, 61.5% and 72.4% of total annual costs in the pre-withdrawal, first and second year post-withdrawal period, respectively.

**Conclusion:**

In the first two years after initiating withdrawal, the total annual costs were decreased compared with the pre-withdrawal period. However, cost reductions were lower in the second year compared with the first year post-withdrawal, primarily due to restarting or intensifying biologics. To support biologic withdrawal decisions, future research should assess the full long-term societal cost impacts, and include all biologics.

Rheumatology key messages

- Consensus upon TNFi withdrawal in JIA patients in clinically inactive disease is currently lacking
- TNFi withdrawal decreases mean annual costs within the first and second year after withdrawal initiation.
- Cost savings were higher in the first compared with the second year after withdrawal initiation.

## Introduction

Juvenile idiopathic arthritis (JIA) is the most common chronic inflammatory disease in childhood, affecting ∼1:1000 children [1, 2]. Besides functional limitations, JIA may result in substantial social and economic burden to patients as well as caregivers [3–9]. The introduction of biologic DMARDs (i.e. biologics) approximately two decades ago has strongly improved the ability to acquire and maintain clinically inactive disease (CID) or low levels of disease activity. This limits long-term physical impairment and comorbidities, and increases quality of life [10, 11]. The recent paradigm shift towards treat-to-target strategies and early aggressive treatment has increased the use of biologics [12]. Currently, TNF-α inhibitors (TNFi) are the most commonly prescribed biologics in JIA [13–15]. Nevertheless, concerns about short-term and long-term side effects, as well as the high costs of biologics compared with conventional synthetic DMARDs (csDMARDs) remain subject to debate [8, 16]. Therefore, safely withdrawing biologics could reduce patient risks as well as healthcare costs. Although recent studies in JIA and in adult rheumatology have identified predictors of successful biologic therapy withdrawal [17–19], consensus on when and how to withdraw (i.e. abrupt discontinuation, spacing the interval or tapering the dose) is not yet available. Furthermore, these withdrawal decisions are complex as 60–83% of patients flare within 12 months of discontinuation [16]. Flares result in additional burden to patients and caregivers, increased healthcare consumption and associated costs [20–22]. Unsurprisingly, the question how medication in JIA can be withdrawn scored second on the Dutch JIA research agenda [23]. Although clinical evidence upon when to withdraw biologic therapy is emerging, research into the cost implications of biologic therapy withdrawal in JIA is lacking. Therefore, this study aims to quantify the hospital-associated costs and accompanying travel costs and productivity losses in JIA patients during their time in CID and during TNFi withdrawal.

## Methods

**Figure 1.**
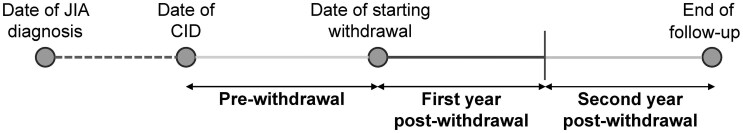
Annual costs were determined in the pre-withdrawal period (starting from date of CID to the date of starting withdrawal) and compared with the post-withdrawal period (starting from the date of starting to withdraw to the end of follow-up). The post-withdrawal period is divided into the first year post-withdrawal and second year post-withdrawal. CID: clinically inactive disease

In this study, the costs of hospital-associated care and accompanying travel costs and productivity losses related to the patient’s JIA were quantified. Annual costs per patient were determined for three time periods: (i) the period of CID (i.e. the ‘pre-withdrawal’ period), represented by the time period from CID until the start of TNFi withdrawal, (ii) the first year after withdrawing TNFi (i.e. ‘first year post-withdrawal’) and (iii) the second year after withdrawing TNFi (i.e. ‘second year post-withdrawal’), as shown in Fig. 1.

### Patient inclusion

This study involved a retrospective analysis of data prospectively collected in the electronic medical records from the paediatric rheumatology department of the tertiary referral centre Wilhelmina Children’s Hospital (Utrecht, the Netherlands). These data were extracted using a previously developed research data platform [24], resulting in a comprehensive set of within-hospital databases connected through a unique, de-identified patient number. The institutional review board classified the use of data from the research data platform as exempt from the Medical Research Involving Human Subjects Act (14/684). This study was conducted according to Good Clinical Practice guidelines and the Declaration of Helsinki [25], and was approved by the ethical committee of the faculty of Behavioural, Management and Social Sciences of the University of Twente (no. 190216). Patients were included if they met all of the following inclusion criteria: age <18 years; treated with TNFi for their JIA in the Wilhelmina Children’s Hospital (Utrecht, the Netherlands); and achieved and maintained CID until an attempt to withdraw their TNFi between 8 April 2011 and 8 April 2022. CID was defined as the date that the treating paediatric rheumatologist assessed that the patient had: (i) no swollen joints, (ii) no joints with both limited range of motion and joint pain, and (iii) a score of zero on the physician’s global assessment scale [26]. The date of CID was obtained from the patient’s electronic medical records. Withdrawal could involve either immediate TNFi discontinuation or a gradual reduction of TNFi intake, either through lengthening the intervals between intake (spacing) or through reducing successive doses (tapering). Within the Wilhelmina Children’s Hospital, spacing is preferred over tapering because spacing decreases the burden of injections to patients. The regular spacing scheme involves 3 months of 1.5 times the regular TNFi treatment interval [27]. If a flare occurs, the patient will either go back to the regular treatment interval or to the longest effective treatment interval. In case the patient remains in CID, the interval of TNFi treatment is increased to two times the normal treatment interval for another 3 months. Finally, when the patient stays in CID after this prolonged spacing interval, the TNFi is stopped as well as any concomitant treatment with csDMARDs, if applicable. The choice to either abruptly discontinue the TNFi (and concomitant DMARD therapy) or space its interval is made in consultation with the patient and parents/caregivers. At our centre, discussions of TNFi withdrawal are initiated after approximately 9 months of clinically inactive disease. In these conversations, it is clarified that it is uncertain whether there will be a difference in flare risk, but that spacing, even when a flare occurs, will give additional evidence about which TNFi treatment interval might be a good alternative to the regular treatment interval, soon after reaching CID again.

Patients were excluded if: (i) a TNFi was prescribed for other reasons than active arthritis, (ii) the patient was diagnosed with systemic JIA, (iii) the patient had a follow-up of <1 year after starting TNFi withdrawal. In addition, patients with <6 months follow-up in the second year post-withdrawal (i.e. 1.5 years from starting TNFi withdrawal) were excluded from the second year post-withdrawal analysis. Data for each included patient were collected from CID until 2 years after withdrawal initiation, 18 years of age or loss to follow-up, whichever came first. No further exclusion criteria were defined regarding the duration of the pre-withdrawal period, which is represented by the time between achieving CID and starting TNFi withdrawal. All patient data were extracted from electronic medical records.

### Resource use

Hospital-associated resource use was extracted on a patient level for the following resource use categories: paediatric rheumatologist consultations, radiology investigations, laboratory testing, hospitalizations and procedures under general anaesthesia (including intra-articular corticosteroid injections). Only hospital-associated resource use that was judged to be JIA-related according to a paediatric rheumatologist was included. In case of doubt, a second paediatric rheumatologist was consulted. Hospital consultations with physicians other than paediatric rheumatologists, for example with ophthalmologists, could not be included in the current analysis as these data were not available.

The following data were extracted regarding medication use during the study period: start date, stop date, dose and accompanying administration interval. Medication included in the analysis were biologics, csDMARDs and corticosteroids (i.e. articular injections and systemic administration). NSAIDs were excluded because these are over-the-counter medications in the Netherlands and therefore their use is not properly recorded. A detailed overview of all inclusion criteria and assumptions made is provided in Supplementary Data S1 (available at *Rheumatology* online).

Subsequently, costs associated with travel and productivity losses for JIA-related hospital visits were approximated on an individual patient level. More specifically, travel distance and time to and from the Wilhelmina Children’s Hospital were estimated using Google Maps and the patients’ four-digit postal code. Productivity loss for the caregiver was estimated based on the travel time for a return trip plus the estimated time spent in the hospital. For hospitalizations and procedures under anaesthesia, time spent in hospital was extracted from the electronic medical records. For regular outpatient rheumatology visits, time spent in the hospital was assumed to be 2 h, which also involves potential time spent on radiology investigations or laboratory testing. It was assumed that one caregiver attended the patient during the hospital visits. Parking time was set equal to the time spent in the hospital. For patients and/or caregivers, telephone consultations were assumed to involve 20 min of lost productivity.

### Resource use costs

All unit costs used were obtained in euros and converted to 2022 values using Dutch consumer price indices. In health economics, discounting can be applied to assign less value to costs (and effects) that occur in the (distant) future [28]. In the current study, no discounting was applied as each of the three consecutive time periods covers a follow-up period of approximately 1 year, and because analysing the future cost impact of biologic therapy withdrawal was beyond the scope of the current study.

The costs per rheumatology visit, per telephone consultation and per hospitalization day were obtained from the Dutch Costing Manual, as well as travel costs and costs of lost productivity of the caregiver [29] (see Table 1). Parking costs were based on hourly tariffs at the Wilhelmina Children’s Hospital [30]. The costs of outpatient visits associated with administering a biologic were derived from the Dutch Healthcare Authority [31]. Costs for laboratory testing, radiology investigations and procedures under anaesthesia were determined by multiplying the costs for each individual test or investigation (as obtained from the Dutch Healthcare Authority [31]) with the frequency at which these were performed. Similarly, medication costs were calculated by multiplying the unit costs of the drug (obtained from the Dutch pharmaceutical list prices [32]) with the dose, duration and frequency of use, as shown in Supplementary Data S1 (available at *Rheumatology* online).

**Table 1.** Description of unit prices used

Category	Unit price	Resource	Explanation or assumption
Rheumatology visit	€112.17	Dutch Costing Manual [28]	Tariff of an outpatient paediatric department visit
Telephone consultation	€56.09	Dutch Costing Manual [28]	Assumed to cost 50% of an outpatient paediatric department visit
Hospitalization day	€696.32	Dutch Costing Manual [28]	Tariff of a nursing day at paediatric department
Administering a biologic	€408.33	Dutch Healthcare Authority [30]	Tariff of administering a biologic during an outpatient visit
Laboratory tests	NA	Dutch Healthcare Authority [30]	Determined for every individual test
Radiology investigations	NA	Dutch Healthcare Authority [30]	Determined for every radiology investigation
Procedures under anaesthesia	NA	Dutch Healthcare Authority [30]	Determined for every procedure, predominantly involves joint injections
Medication	NA	Dutch pharmaceutical list prices [31]	Determined for every drug per prescribed dose^a^
Dispensing fee medication	€6.00	Dutch Costing Manual [28]	Fee per medication prescription, repeated every 90 days
Travel costs per kilometre	€0.21	Dutch Costing Manual [28]	—
Lost productivity of the caregiver per hour	€38.36	Dutch Costing Manual [28]	—
Parking costs per hour	€1.80	Wilhelmina Children’s Hospital [29]	Hourly parking rate at Wilhelmina Children’s Hospital

This table provides a description of the cost categories included in the analysis, the unit price used for each cost category, a reference to the source where the unit price was obtained from, and if applicable, an explanation or assumption regarding how these costs were determined. ^a^An overview of all drug costs is included in Supplementary Data S2, available at *Rheumatology* online. NA: not applicable.

### Analysis

All analyses were performed in R version 4.0.3 (R Foundation for Statistical Computing, Vienna, Austria). The total costs per patient were determined by multiplying the patient’s resource use with the accompanying unit costs. The costs were reported as the mean total annual costs in euros per patient per time period (i.e. pre-withdrawal, first year post-withdrawal, second year post-withdrawal) and subdivided into the different cost categories. One-way sensitivity analyses were used to assess the impact of varying all types of cost inputs with +25% and −25% to account for uncertainty in cost inputs, including TNFi price fluctuations, on the cost differences between the pre-withdrawal and both post-withdrawal periods. Analyses were performed for each cost category (i.e. medication dispensing and use, paediatric rheumatologist consultations, telephone consultations, radiology investigations, laboratory testing, hospitalizations, procedures under general anaesthesia, administering biologics, lost productivity, parking and travel) as well as per type of TNFi, and were visualized using tornado diagrams.

Differences in annual costs between (i) the pre-withdrawal period, (ii) the first year post-withdrawal, and (iii) the second year post-withdrawal were calculated using Kruskal–Wallis and Mann–Whitney *U* tests, as appropriate. *P*-values were corrected for multiple testing according to Benjamini and Hochberg [33]. All comparisons were two-sided and *P*-values <0.05 were considered statistically significant.

## Results

**Figure 2.**
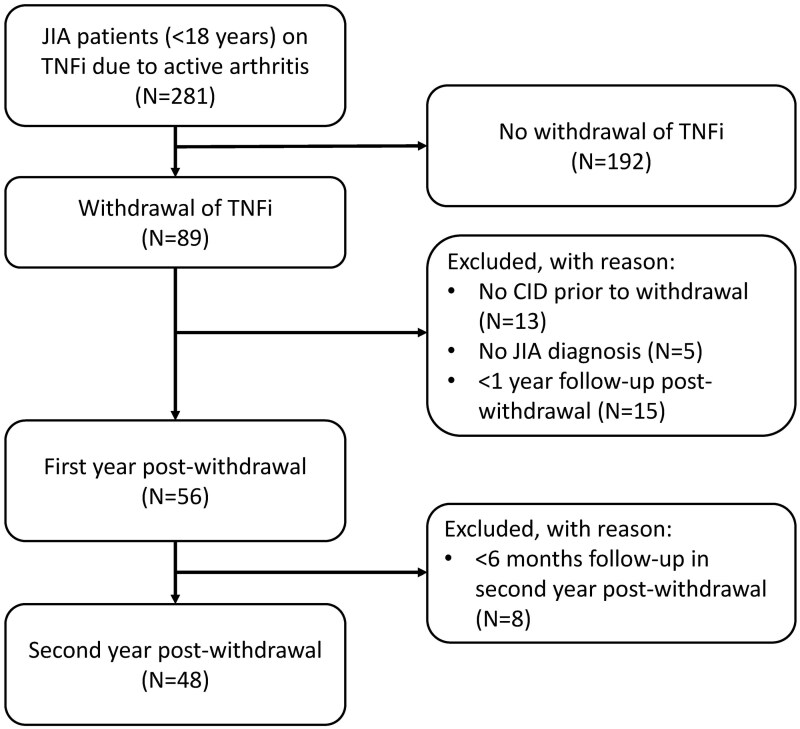
Flowchart of patient inclusion. CID: clinically inactive disease; TNFi: TNF-α inhibitors

Out of the 281 patients who were treated with TNFi due to active arthritis between 8 April 2011 and 8 April 2022 in the Wilhelmina Children's Hospital, 56 patients were included in the analysis (Fig. 2). Of these 56 patients, 31 (i.e. 55%) were girls (Table 2). The median age at CID (i.e. at the start of the study) was 10.2 years (interquartile range [IQR]: 6.5–13.0). Eight out of 56 patients were excluded from the second year post-withdrawal analysis because they had a follow-up of <1.5 years after starting TNFi withdrawal. Patients in the abrupt discontinuation group (*n* = 26) were significantly younger (at JIA diagnosis, CID and at the start of withdrawal) compared with patients who spaced the treatment interval (*n* = 30). In the abrupt discontinuation group, 16 patients restarted treatment within the first year due to a disease flare, five within the second year and five did not restart biologic treatment within the study period. For patients that increased their treatment interval, 13 patients flared within the first year and required a dose increase, 12 within the second year and five did not require restarting biologic treatment within the study period. Median time to restart biologic treatment was 8 months (IQR 6–14 months). One patient was treated with systemic corticosteroids while restarting biologic treatment.

**Table 2.** Characteristics of the patient population

Characteristics	Total (*n* = 56)
Age at symptoms onset, median (IQR), years	6.9 (2.7–10.5)
Age at JIA diagnosis, median (IQR), years	7.5 (3.9–11.5)
Age at starting biologic therapy, median (IQR), years	9.3 (6.0–12.2)
Age at CID, median (IQR), years	10.2 (6.5–13.0)
Age at starting to withdraw, median (IQR), years	11.1 (8.6–14.5)
Duration in CID prior to withdrawal, median (IQR), years	1.0 (0.8–1.4)
Gender, *n* (%)	
Female	31 (55.4)
Male	25 (44.6)
JIA subtype, *n* (%)	
Enthesis related arthritis	7 (12.5)
Extended oligo-articular JIA	9 (16.1)
Persistent oligo-articular JIA	15 (26.8)
Juvenile psoriatic arthritis	5 (8.9)
RF-negative polyarticular JIA	16 (28.6)
RF-positive polyarticular JIA	4 (7.1)
TNFi therapy, *n* (%)	
Adalimumab	30 (53.6)
Etanercept	25 (44.6)
Golimumab	1 (1.8)
Concomitant medication, *n* (%)	
Methotrexate	41 (73.2)
Leflunomide	3 (5.4)
Systemic corticosteroid	1 (1.8)
TNFi course, *n* (%)	
First	51 (91.1)
Second	5 (8.9)
ANA status, *n* (%)	
Positive	25 (44.6)
Negative	30 (53.6)
Missing	1 (1.8)
RF status, *n* (%)	
Positive	5 (8.9)
Negative	40 (71.4)
Missing	11 (19.6)
HLA B27 status, *n* (%)	
Positive	13 (23.2)
Negative	24 (42.9)
Missing	19 (33.9)

CID: clinically inactive disease; IQR: interquartile range; TNFi: TNF-α inhibitors

### Total group

The mean annual costs of hospital-associated care and accompanying travel costs and productivity losses for JIA were €9165/patient (95% CI: €8343, €10 047) in the pre-withdrawal period and reduced to €5063/patient (95% CI: €4339, €5808) in the first year post-withdrawal (*P* < 0.05) and to €6569/patient (95% CI: €5507, €7582) in the second year post-withdrawal (*P* < 0.05) (Table 3 and Supplementary Data S3, available at *Rheumatology* online). The mean follow-up in these three periods was 428, 365 and 353 days, respectively. Of these costs, €834/patient (9.1%), €1182/patient (23.3%) and €1317/patient (20.1%), respectively, was attributable to travel costs and/or productivity losses. For 52 patients (92.9%) the annual costs reduced in the first year post-withdrawal compared with the pre-withdrawal period, ranging from −€92 to −€19 155. In the remaining four patients, annual costs increased by €51/patient to €1393/patient, primarily attributable to restarting biologic therapy. Three out of four patients abruptly discontinued their TNFi and one patient increased the TNFi treatment interval. In the second year post-withdrawal, the annual costs reduced for 72.9% of the patients (*n* = 35) compared with the pre-withdrawal period. Of these patients, 42.9% (*n* = 15) had abruptly discontinued their TNFi (cost reductions ranging from −€18 734 to −€625) and 57.1% (*n* = 20) had increased their treatment interval (cost reductions ranging from −€9867 to −€455). For the remaining 27.1% of patients (*n* = 13), annual cost increased by between €279 and €7423 for eight patients who abruptly discontinued their TNFi and from €86 to €10 672 for five patients who had increased their TNFi treatment interval.

**Table 3.** The annual costs per patient in the three periods (pre-withdrawal, first year post-withdrawal and second year post-withdrawal) for all patients (i.e. the abrupt discontinuation group and spacing group), specified according to the different cost categories

	Pre-withdrawal, absolute value, mean (95% CI) (*n* = 56)	First year post-withdrawal, absolute value, mean (95% CI) (*n* = 56)	Difference *vs* pre-withdrawal, %	Second year post-withdrawal, absolute value, mean (95% CI) (*n* = 48)	Difference *vs* pre-withdrawal, %
Medication	€7398 (€6633, €8296)	€3112 (€2606, €3711)	−57.9	€4757 (€3866, €5751)	−35.7
Rheumatology visits and TC	€1217 (€1097, €1351)	€1349 (€1193, €1511)	10.8	€1190 (€1007, €1400)	2.2
Radiology investigations	€122 (€80, €167)	€159 (€96, €228)	30.3	€187 (€130, €246)	53.3
Laboratory testing	€195 (€134, €290)	€145 (€109, €190)	−25.6	€161 (€96, €281)	−17.4
Hospitalization	€230 (€0, €663)	€290 (€33, €707)	26.1	€261 (€19, €630)	13.5
Procedures under anaesthesia	€2 (€0, €7)	€8 (€0, €20)	300.0	€14 (€0, €37)	600.0
Total costs per patient	€9165^a^ (€8362, €10 070)	€5063^b^ (€4322, €5849)	−44.8	€6569^c^ (€5426, €7739)	−28.3

a–c Of the total costs reported in the bottom row of this table, ^a^9.1%, ^b^23.3% and ^c^20.1% are associated with travel costs and productivity losses. TC: telephone consultations.

For all three time periods, the majority of costs consisted of medication costs, which comprised 80.7%, 61.5% and 72.4% of total costs for the pre-withdrawal period, first year post-withdrawal period and second year post-withdrawal period, respectively (see Table 3, and Supplementary Data S4, available at *Rheumatology* online for a detailed overview). The remaining costs were primarily attributable to paediatric rheumatologist visits.

### Sensitivity analyses

Varying individual cost inputs per cost category by +25% and −25% indicated that medication cost had the biggest impact on the difference in mean annual costs between the pre-withdrawal, first year post-withdrawal and second year post-withdrawal period. Costs of adalimumab and etanercept treatment primarily influenced the cost differences (Supplementary Data S5, available at *Rheumatology* online). Nevertheless, the sensitivity analyses demonstrated that changing the cost inputs with −25% and +25% still resulted in lower mean annual total costs in the first year post-withdrawal and second year post-withdrawal when compared with the pre-withdrawal period.

## Discussion

The study provides unique insights into the hospital-associated costs and accompanying travel costs and productivity losses in patients with JIA before and after initiating TNFi withdrawal. The findings indicate that TNFi withdrawal was associated with lower mean annual costs of JIA-related care in the first two years after withdrawal initiation compared with the pre-withdrawal period. Costs were higher in the second year post-withdrawal compared with the first year post-withdrawal, attributable to patients re-starting or intensifying biologic therapy, but costs were lower compared with the pre-withdrawal period.

Overall, medication costs contributed to 61.5–80.7% of the total costs of JIA-related care in our study population. The current study, however, included a specific subset of biologic users in CID who start with discontinuation or spacing. Nevertheless, previous studies also reported that medication costs contributed to 41–85% of the costs of JIA-related care [9, 34–38]. More specifically, the annual costs reported in the current study are in line with previous research, e.g. the mean annual cost of etanercept treatment is €12 478/patient in the Netherlands [39]. However, reported costs are highly dependent on disease activity, whether patients are on biologic therapy, the costing methodology used and the country in which the study is performed [34, 37, 40].

Previous research has indicated that higher treatment costs are particularly present within the first weeks after JIA diagnosis due to a higher frequency of laboratory tests, radiology investigations and rheumatology visits [7, 9]. In our study, none of the included patients achieved CID within 3 months following their JIA diagnosis, indicating that this period with high healthcare consumption was not included in the pre-withdrawal period in the current study and therefore did not affect the study's results.

### Strengths

This is the first study to investigate JIA-associated costs during a period of CID and during the first and second year after TNFi withdrawal, using data prospectively collected in JIA patients over a mean follow-up period of 3 years. Although the evidence from this study needs to be combined with evidence about the impact of biologic withdrawal on safety, efficacy and quality of life, this is an important step in formulating evidence-based treatment recommendations regarding biologic withdrawal in JIA patients in CID.

### Limitations

The current study was subject to limitations. First, only patients on TNFi biologics were included in the analysis as this type of biologics is the most commonly used (i.e. 71% of JIA patients on biologics) [8, 16]. However, in future studies, it is advisable to also incorporate other types of biologics.

Second, only 56 out of the 281 patients evaluated met the inclusion criteria, which limits the generalizability of the findings. The main reason for excluding patients was that there was no attempt to withdraw biologic therapy in the time period for which data were available. Although comparing the costs of patients who did *vs* those who did not withdraw TNFi was considered, this would have resulted in selection bias. More specifically, patients may not have withdrawn TNFi because they had active disease, because of difficulties in reaching inactive disease or because of a history of flaring after TNFi withdrawal.

Third, costs could have been missed due to a lack of data. In this study, data were missing regarding within-hospital physician visits other than visits to the paediatric rheumatologist, as these data were only available up to 12 December 2018 and could therefore not be included in the current analysis. Nevertheless, a previous study showed that costs of other within-hospital physician visits account for approximately one-third of all outpatient hospital visit costs [7]. Thus, it is unlikely that including these costs would have changed the findings of the current study. In addition, data regarding JIA-related care received by patients outside the Wilhelmina Children’s Hospital and the corresponding productivity losses of the caregiver and the patient could not be included. We do acknowledge that an analysis of the full cost impact of JIA should preferably also incorporate costs of care that patients receive in other treatment centres. The impact of JIA on total costs from a societal perspective is currently being evaluated in a large prospective, multicentre, international collaborative study into JIA management strategies, conducted in Canada and the Netherlands, named UCAN CAN-DU (https://www.ucancandu.com/) [41].

Fourth, medication costs are based on the tariffs reported by the National Healthcare Institute and might not correspond with the actual contract prices negotiated between the pharmaceutical company and the Wilhelmina Children’s Hospital. Also, price fluctuations could not be included in the analysis, but are important for biologics especially since biosimilars have entered the market. However, the sensitivity analysis indicated that this uncertainty is highly unlikely to affect the overall conclusions.

### Impact and generalizability of study findings

Due to the lack of guidance regarding tapering/stopping TNFi (or biologic therapies in general), major variation in tapering/stopping strategies is observed between treatment centres and between clinicians. In our centre, it is common practice to discontinue TNFi and concomitant treatment simultaneously. Nevertheless, four patients with concomitant treatment continued methotrexate therapy after discontinuation of their TNFi. Although two of these patients did not require restarting biologic therapy within the study period, numbers are too small to reach any meaningful conclusions. Although the current study is a single-centre study, which may limit the generalizability of study findings, it was conducted in the largest paediatric rheumatology treatment centre in the Netherlands. Thereby, this study provides useful insights into currently applied withdrawal strategies. However, due to the impact of aspects like disease status, medication use and time elapsed since JIA diagnosis on the total annual costs, caution is required when comparing the results of the current study with other countries as JIA treatment strategies and guidelines might differ between countries. For example, in contrast to other countries, patients in the Netherlands do not have a waiting period before starting JIA treatment [42], which might positively affect treatment efficiency and thereby lead to lower healthcare resource use and accompanying costs. Furthermore, the reported differences in costs are not only explained by differences between treatment strategies and guidelines but also by differences in unit costs between countries. Besides, the results in this study are based on the analysis of 56 patients in whom it was attempted to taper or stop biologic therapy. Although it is likely that comparable cost savings can be achieved among the 33 patients who attempted to taper or stop but who were excluded from this analysis for a variety of reasons, the current analysis also excluded 192 patients because they did not undergo a withdrawal attempt between 8 April 2011 and 8 April 2022. When consensus will be reached about when and how to withdraw TNFi (or biologic therapies in general), the number of patients in whom withdrawal is attempted will likely increase, thereby also increasing cost savings in the first two years after initiating withdrawal.

### Implications for further research

The ultimate goal of JIA treatment is to reduce the patient’s symptoms, restore their physical and psychological functioning, and thereby prevent or limit long-term joint damage and disability [12, 43]. Part of this treatment might be the withdrawal of biologics after reaching CID, but evidence-based guidelines regarding when and how to withdraw biologics, as well as their impact on costs, is currently lacking. Therefore, future studies are needed to capture the implications of different strategies of biologic therapy withdrawal in terms of treatment effectiveness (including disease activity and flare rate), costs and quality of life. The relatively small number of patients in the current study unfortunately did not allow such a subgroup analysis. A study into the quality of life of adult rheumatoid arthritis patients during biologics withdrawal has already shown that biologic therapy withdrawal is cost-saving, but that it decreases the quality of life compared with standard care [44]. The influence of biologic therapy withdrawal on quality of life in JIA patients is currently being investigated in the UCAN CAN-DU project [41]. Finally, the study focused on the first two years after starting TNFi withdrawal. However, future studies should investigate the long-term cost impact of biologic withdrawal strategies, and incorporate other biologics than TNFi only.

## Conclusion

In summary, the results of the current study indicate that withdrawing TNFi in JIA patients <18 years in CID on TNFi is associated with lower mean annual costs within the first and second year after starting to withdraw TNFi compared with the pre-withdrawal period. The greatest cost reductions are achieved within the first year after starting TNFi withdrawal. Compared with this first year, the cost reductions decrease within the second year after starting TNFi withdrawal, primarily due to restarting or intensifying biologic therapy. Medication costs were found to be the main cost driver in all periods and for all patients, regardless of whether the TNFi was abruptly discontinued or spaced first. The cost reductions in both these withdrawal strategies did, however, not differ significantly in the first and second year post-withdrawal compared with the pre-withdrawal period. To capture the full cost impact of biologic therapy withdrawal in JIA, future research should also incorporate its impact on medical consumption outside the hospital, a longer follow-up period, and other types of biologics.

## Supplementary Material

kead688_Supplementary_Data

## Data Availability

The data that support the findings of this study are available upon reasonable request and by contacting the corresponding author, but restrictions apply to the availability of these data, which were used under licence for the current study, and so are not publicly available. Data are however available from the authors upon reasonable request and with the permission of J.S.
