# Correction to: Quantifying hospital-associated costs, and accompanying travel costs and productivity losses, before and after withdrawing TNF-α inhibitors in juvenile idiopathic arthritis

**DOI:** 10.1093/rheumatology/keaf405

**Published:** 2025-08-25

This is a correction to: Anna A Florax, Martijn J H Doeleman, Sytze de Roock, Naomi van der Linden, Ellen Schatorjé, Gillian Currie, Deborah A Marshall, Maarten J IJzerman, Rae S M Yeung, Susanne M Benseler, Sebastiaan J Vastert, Nico M Wulffraat, Joost F Swart, Michelle M A Kip, for UCAN-CAN DU and UCAN CURE Consortia, Quantifying hospital-associated costs, and accompanying travel costs and productivity losses, before and after withdrawing TNF-α inhibitors in juvenile idiopathic arthritis, *Rheumatology*, Volume 63, Issue SI2, September 2024, Pages SI143–SI151, https://doi.org/10.1093/rheumatology/kead688

In the originally published version of the manuscript there was a formatting error in the last name of the eighth co-author. This has been emended in the paper to read: “Maarten J IJzerman”.

