# Supplementary material for: Quantifying hospital-associated costs, and accompanying travel costs and productivity losses, before and after withdrawing TNF-α inhibitors in juvenile idiopathic arthritis
Source: Rheumatology (Oxford). 2023 Dec 20;63(SI2):SI143–51. doi: 10.1093/rheumatology/kead688 (PMC11381678; doi:10.1093/rheumatology/kead688)
Supplement: kead688_Supplementary_Data [file kead688_supplementary_data.docx]

Group members for UCAN CAN-DU:

Rae S.M Yeung

Nico Wulffraat

Susanne M. Benseler

Joost F. Swart

Sebastiaan J. Vastert

Marinka Twilt

Deborah A. Marshall

Joseph Cafazzo

Group members for UCAN CURE:

Rae S.M Yeung

Susanne M. Benseler

Deborah A. Marshall

Joseph Cafazzo

Maarten J. IJzerman

Nico Wulffraat

Joost F. Swart

Sebastiaan J. Vastert

Marinka Twilt

## Supplementary data S1 - Inclusion of hospital-associated resource use and corresponding costs

For the analysis, hospital-associated resource use (including medication use) of the patient within the follow-up period of the patient is included. All costs were corrected for inflation using the Consumer Price Indices (CPI) from Statistics Netherlands and the following formula:


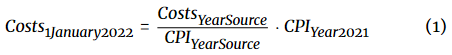


**Radiology investigations**

Radiology investigations with the status ’Completed’, as recorded in the Research Data Platform, were included in the analysis. The costs were obtained from the Dutch Healthcare Authority (1), and were not included in the analysis if:

- The investigation was performed for (clinical) study purposes.
- The costs of the investigation were already included in another procedure performed on the same day.
- The investigation was judged to be unrelated to JIA by a paediatric rheumatologist.

The included radiology investigations and their frequencies were given in Supplementary Figure S1.A


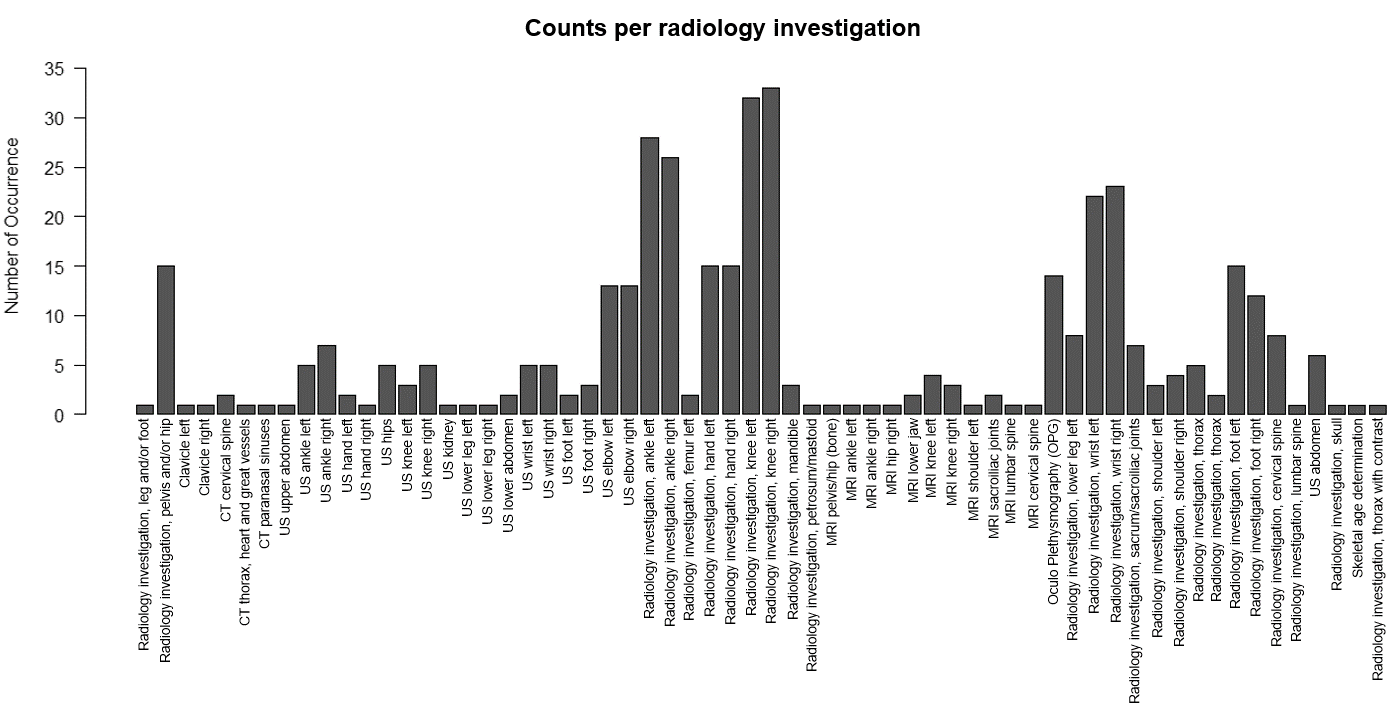


**Supplementary Figure S1.A The counts per included radiology investigation during the follow-up period**

**Laboratory testing**

Laboratory tests with the status 'Completed', as recorded in the Research Data Platform, were included in the analysis. The costs were obtained from the Dutch Healthcare Authority (1), and were not included in the analysis:

- The test was already included in another investigation that day, such as Mean Platelet Volume, which reported for free when a thrombocyte count is performed.
- The test was conducted for clinical studies.
- The test was clearly unrelated to JIA, such as a codfish allergy test.

The included laboratory tests and their frequency were given in Supplementary Figure S1.B.


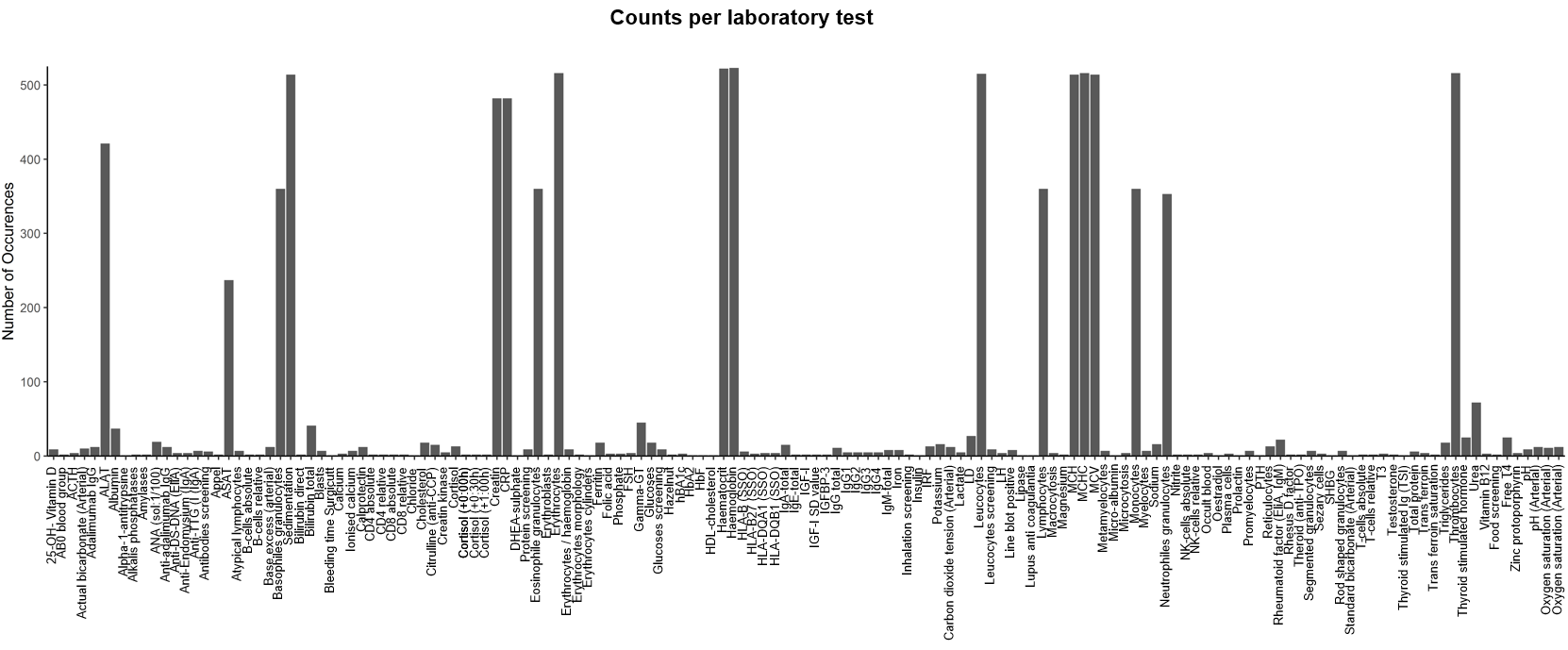


**Supplementary Figure S1.B The counts per included laboratory tests during the follow-up period**

**Rheumatology visits and telephone consultations**

Only hospital visits to and telephone consultations with the department of Paediatric Rheumatology and with the status ’Completed’, as recorded in the Research Data Platform, were included in the analysis. The following choices were made:

- Rheumatology visits with the label 'operational' were manually checked in the Electronic Medical Record to determine which activity has been conducted and if additional costs should be added.
- Appointments which were not labelled as telephone or in-person were assumed to be in-person.

The costs for rheumatology visits were based on the reference prices for outpatient visits at the paediatric department, as reported in the Dutch Costing Manual and additional costs were added for travel expenses, parking costs and labour productivity losses (2,3).

**Hospitalisation**

For hospitalisations, it was decided on an individual patient level by a paediatric rheumatologist whether the visit was directly related to JIA care. Included hospitalisations were: bDMARD intravenous administration and hospitalisation after an intra-articular injection under sedation. The corresponding costs were determined using the reference prices for inpatient days at the paediatric department, as documented by the Dutch Costing Manual, plus additional costs for travel expenses, parking costs and labour productivity losses (2,3). For the bDMARD injection, the reference price as reported by the Dutch Healthcare Authority was used (1).

**Procedures under anaesthesia**

For procedures under anaesthesia, it was decided on an individual patient level by a paediatric rheumatologist whether the visit was directly related to JIA care. Ultimately, only intra-articular injections under sedation were included within the follow-up period. The costs for this procedure were based on the tariffs of the Dutch Healthcare Authority (€229.51) plus additional costs for travel expenses, parking costs and labour productivity losses (1–3). If a procedure under anaesthesia is accompanied by a day-care hospitalisation, the labour productivity losses costs, travel expenses and parking costs were charged once.

The number of intra-articular injections was determined from the operating room database and a supplemental database, which separately reported the administration of the joint injections. The intra-articular injections per patient within the follow-up period were excluded if the date of administration was equal to:

- The date of an intra-articular injection in the operating room to prevent double-counting. The costs for intra-articular steroid injections were included as costs of 'Procedures under anaesthesia'.
- The date of hospitalisation to prevent double-counting. The costs for intra-articular steroid injections were included in the hospitalisation category.

**Medication**

Medication use was manually extracted from the Electronic Medical Record. The following medicines were included in the analysis:

- abatacept
- adalimumab
- anakinra
- azathioprine
- Baricitinib
- canakinumab
- cellcept (mycophenolic acid)
- certolizumab
- ciclosporin
- colchicine
- cyclophosphamide
- dexamethasone
- etanercept
- golimumab
- hydrocortisone
- hydroxychloroquine
- infliximab
- leflunomide
- methotrexate
- prednisolone
- rituximab
- sulfasalazine (salazopyrin)
- sarilumab
- secukinumab
- tocilizumab
- tofacitinib
- ustekinumab

All the dose changes or medicine changes were reported. The included medicines and their frequencies were given in Supplementary Figure S1.C. The medicine names as stated above were matched to their pharmaceutical medicine name which is dispensed by the pharmacy to calculate the exact costs of the dispensed medicine. The costs were calculated using the following formula:


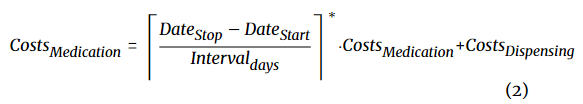


The number of administrations is rounded up to avoid underestimation. The dispensing costs were charged when the patient switches to another medicine/type/dose and were repeated every ninety days (2).


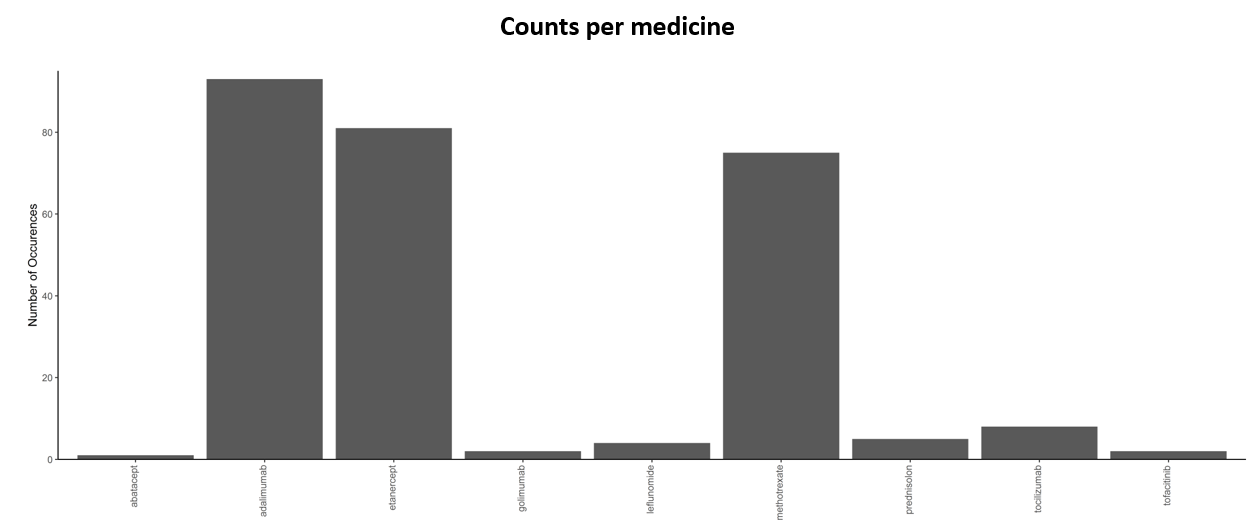


**Supplementary Figure S1.C The counts per medicine during the follow-up period.**

## Supplementary data S2 – Costs of DMARDs

**Supplementary Table S1 Costs of DMARDs** (4)

| DMARD | Pharmaceutic name | Costs | Range |
| --- | --- | --- | --- |
| **Abatacept** | Orencia disposable injection pen 125 mg=1 ml (125mg/ml) | €270.89 |  |
| **Adalimumab** | Adalimumab injection fluid 0.8 ml (50 mg/ml) | €328.05 | (€325.60 - €328.87) |
|  | Adalimumab disposable injection pen 20 mg=0.2 ml (100 mg/ml) | €237.42 |  |
|  | Adalimumab disposable injection pen 40 mg=0.8 ml (50 mg/ml) | €328.05 | (€325.60 - €328.87) |
| **Etanercept** | Enbrel injection flacon 25 mg + solvents 1 ml + attachments | €94.67 |  |
|  | Enbrel disposable injection pen 50 mg=1 ml (50 mg/ml) | €165.91 |  |
|  | Enbrel 14 mg injection fluid for subcutaneous use | €73.12 |  |
|  | Etanercept injection flacon 10 mg + attachments | €50.30 |  |
| **Golimumab** | Golimumab injection pen 50 mg=0.5 ml (100 mg/ml) | €993.58 |  |
| **Leflunomide** | Leflunomide tablet 10 mg | €1.03 |  |
| **Methotrexate** | Methotrexate injection pen 7.5 mg=0.3 ml (25 mg/ml) | €8.23 |  |
|  | Methotrexate injection pen 10 mg=0.2 ml (50 mg/ml) | €10.98 | (€8.23-€13.72) |
|  | Methotrexate disposable injection pen 15mg=1.5ml (10mg/ml) | €12.84 |  |
|  | Methotrexate tablet 2.5 mg | €0.17 |  |
|  | Methotrexate tablet 10 mg | €0.80 |  |
| **Prednisolone** | Prednisolone tablet 5mg | €0.05 |  |
|  | Prednisolone tablet 20mg | €0.12 |  |
| **Tofacitinib** | Tofacitinib tablet 5 mg | €13.51 |  |
| **Tocilizumab** | Tocilizumab infuse solution 10 ml (20 mg/ml) | €358.34 |  |
|  | Tocilizumab infuse solution 20 ml (20 mg/ml) | €716.68 |  |
|  | Tocilizumab disposable injection pen 162 mg=0.9 ml (180 mg/ml) | €256.55 |  |

## Supplementary data S3 – Annual total costs per period


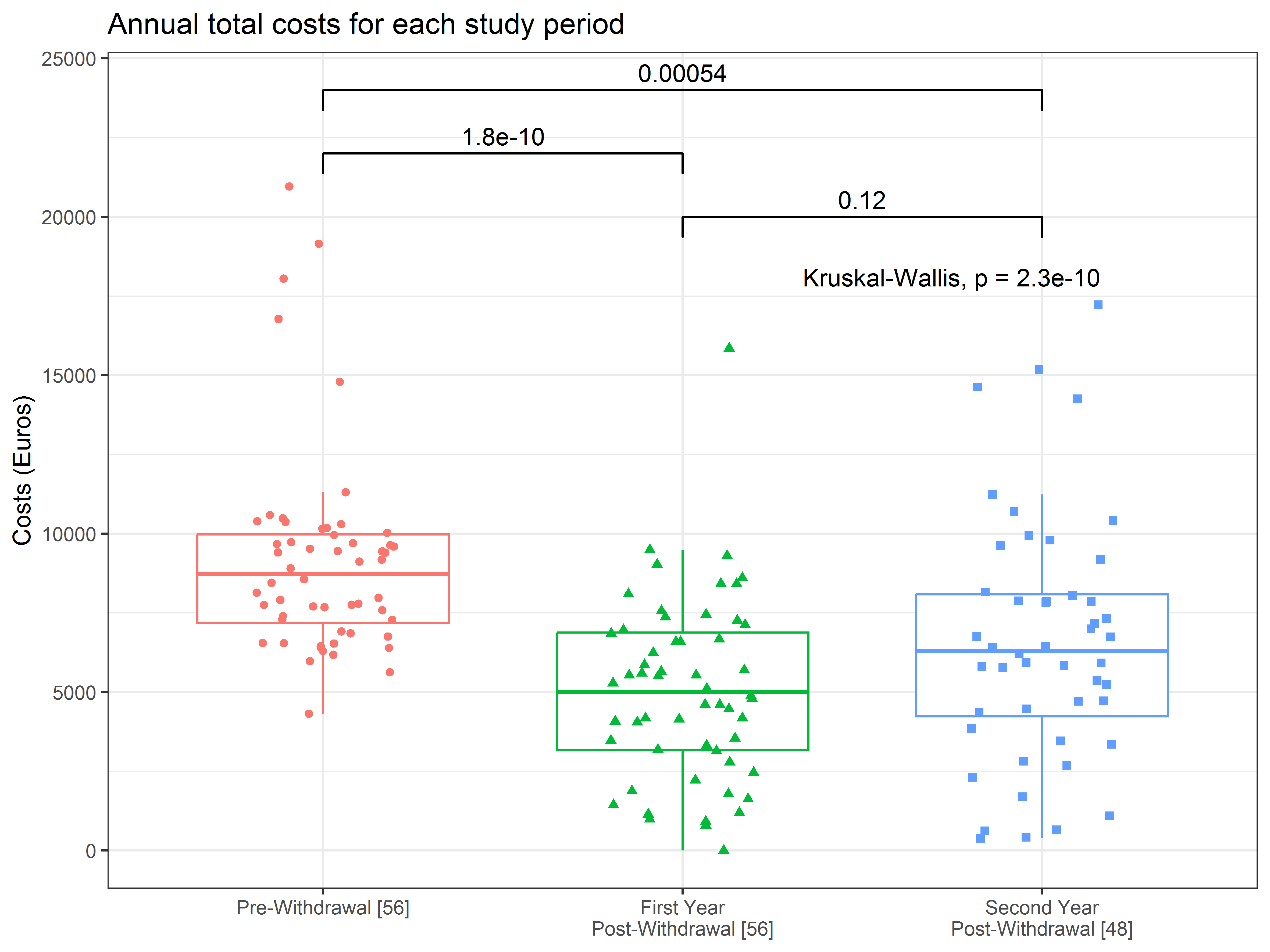


**Supplementary Figure S2 The annual costs per period (pre-withdrawal, first year post-withdrawal, and second year post-withdrawal) for the total study population. The number between squared brackets represents the number of patients in each group.**

## Supplementary data S4 - Annual costs per category


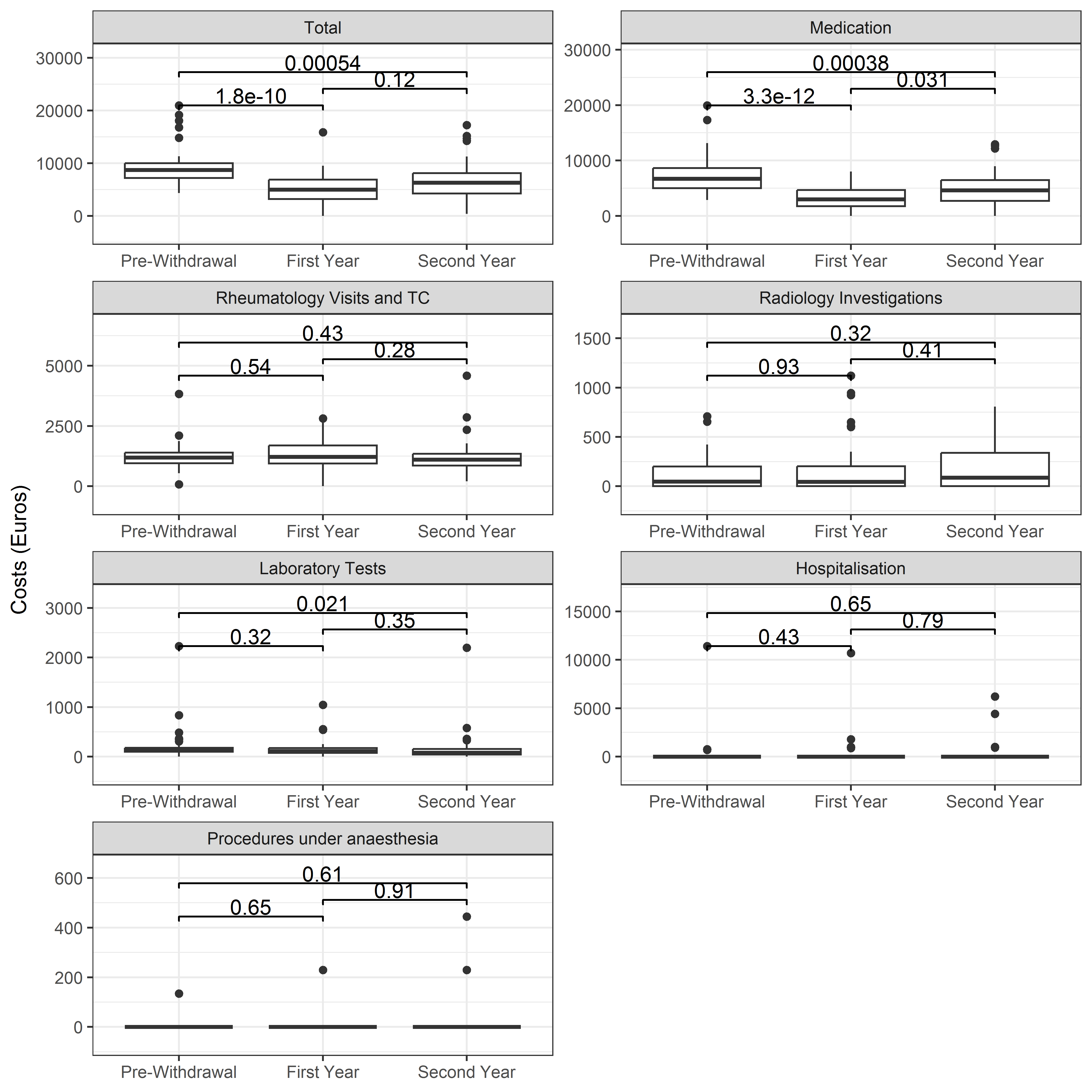


**Supplementary Figure S3 Annual costs in each study period pre-withdrawal, first year post-withdrawal, and second year post-withdrawal) per cost category. Numbers represent p-values, corrected for multiple testing. TC= telephone consultation.**

## Supplementary data S5 – Sensitivity analyses

The impact of varying cost inputs on the cost differences between the pre-withdrawal period and first year post-withdrawal and second year post-withdrawal are presented in Supplementary Figure S4 As the tornado diagrams illustrate, varying the medication costs by ±25% resulted in maximum changes in cost differences of 26.0% (i.e. ±€1,068) and 25.0% (i.e. ±€657) with respect to the base case cost differences (i.e. €4,102 and €2,595), between the pre-withdrawal period and first year post-withdrawal and the pre-withdrawal period and the second year post-withdrawal, respectively. For the cost difference between the pre-withdrawal period and the first year post-withdrawal, varying the hourly labour productivity costs and the laboratory costs resulted in the second (0.4%) and third greatest change (0.3%), with respect to the base case cost difference. For the cost difference between the pre-withdrawal period and the second year post-withdrawal, the variation of the cost inputs for the hourly labour productivity and hospitalisation resulted in respectively the second greatest change (0.8%) and third greatest change (0.6%) with respect to the base case cost difference.

However, in all sensitivity analyses, total costs are lower in both withdrawal periods compared to the pre-withdrawal period. To test the robustness of this result, it was determined what reduction in medication costs would be required to make the withdrawal of medication no longer cost-saving. It was found that the total medication costs should reduce by 96% (i.e. from €7,398 to €488 within the pre-withdrawal period, and from €3,112 to €217 within the first year post-withdrawal) to make the withdrawal of medication no longer cost-saving. In the second year post-withdrawal, total costs were found to be lower compared to the pre-withdrawal period regardless of the reduction in medication costs.

1. **Sensitivity Analysis – Pre-withdrawal vs. First Year**


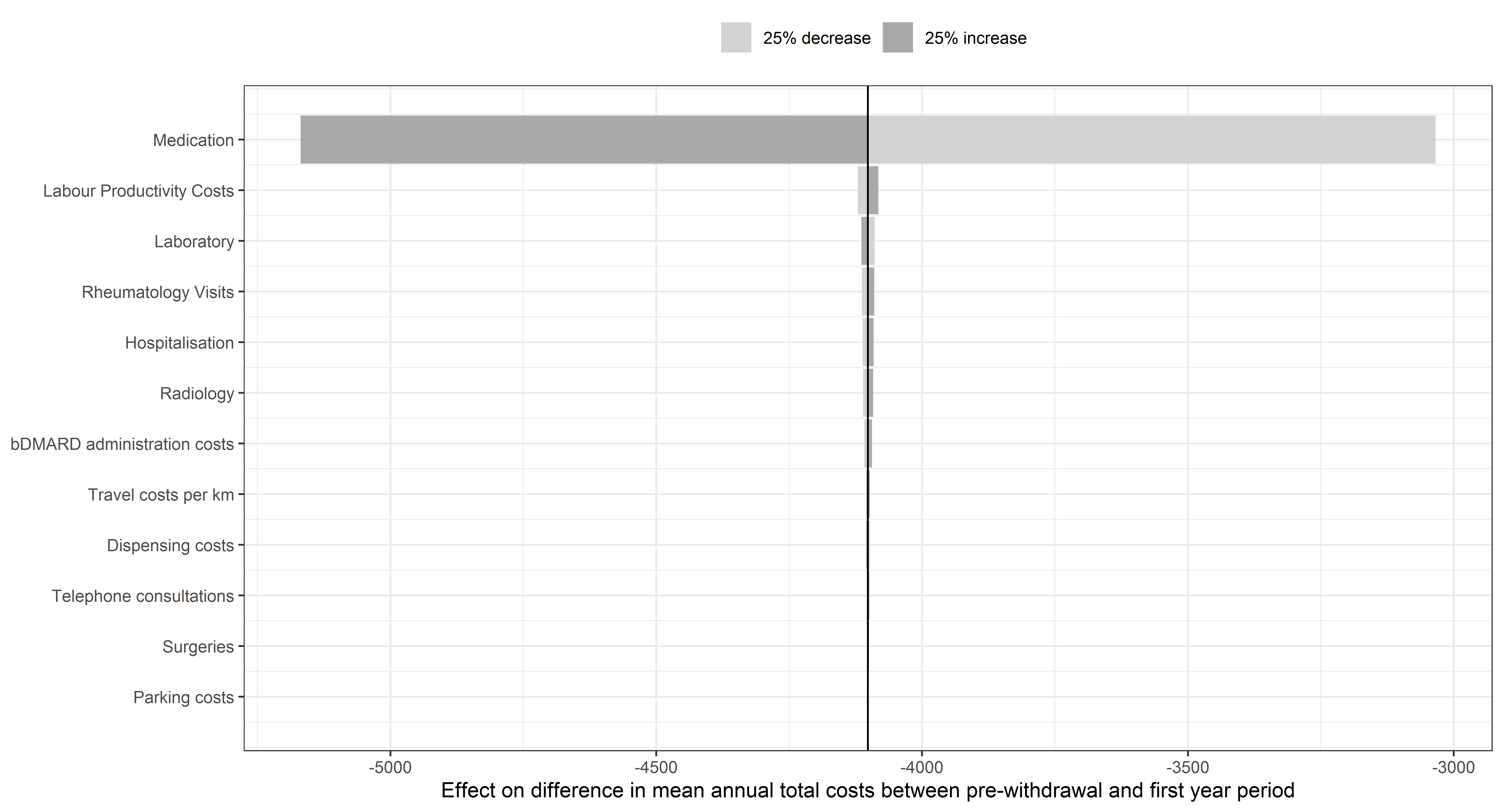


1. **Sensitivity Analysis – Pre-withdrawal vs. Second Year**


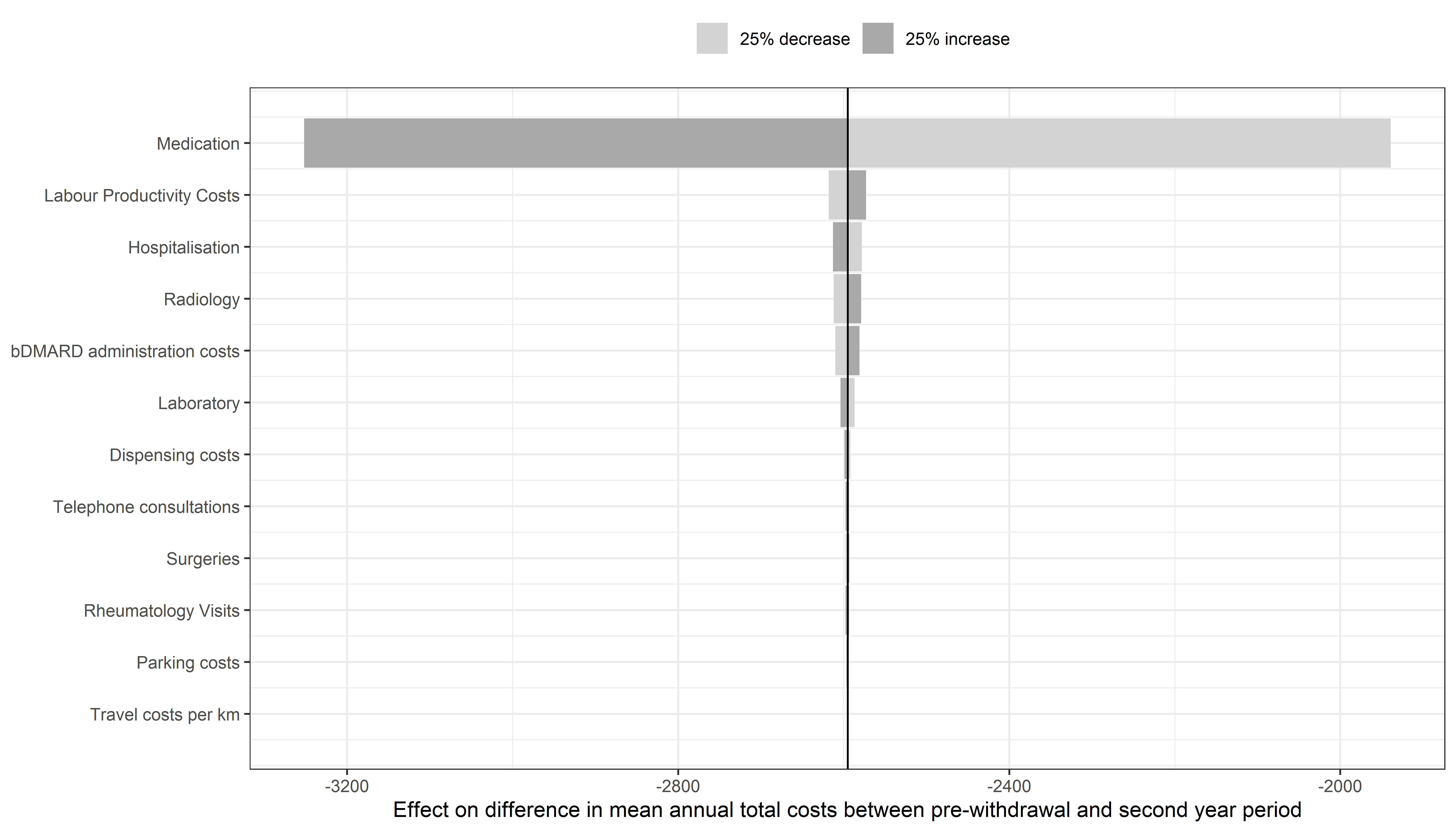


**Supplementary Figure S4 The results of the sensitivity analysis of the different categories of cost inputs. A) the effect of varying the cost inputs per category by [-25% (light grey), +25% (dark grey)] on the mean annual total cost difference between the pre-withdrawal period and the first year post-withdrawal. B) the effect of varying the cost inputs per category by [-25% (light grey), +25% (dark grey)] on the mean annual total cost difference between the pre-withdrawal period and the second year post-withdrawal..**

The impact of varying the costs of individual DMARDs on the cost differences between the 1) pre-withdrawal period and 2a) first year post-withdrawal and 2b) second year post-withdrawal are presented in Supplementary Figure S5. As shown in the tornado diagrams, adalimumab is the DMARD with the highest impact on the total cost differences between the pre-withdrawal and both post-withdrawal periods. For the cost difference between the 1) pre-withdrawal period and 2a) first year post-withdrawal, varying the costs by ±25% per DMARD simultaneously results in a change of 16.0%, 8.9% and 1.3% with respect to the cost difference in the base case (i.e. €4,102) for adalimumab, etanercept and golimumab, respectively. For the cost difference between the 1) pre-withdrawal period and 2b) second year post-withdrawal, varying the costs by ±25% per DMARD simultaneously results in a change of 19.6%, 8.5% and 1.4% with respect to the cost difference in the base case (i.e. €2,595) for adalimumab, etanercept, and tocilizumab, respectively. Despite altering cost inputs for each DMARD type with ±25%, DMARD withdrawal still resulted in cost savings compared to the pre-withdrawal period.

1. **Sensitivity Analysis – Pre-withdrawal vs. First Year**


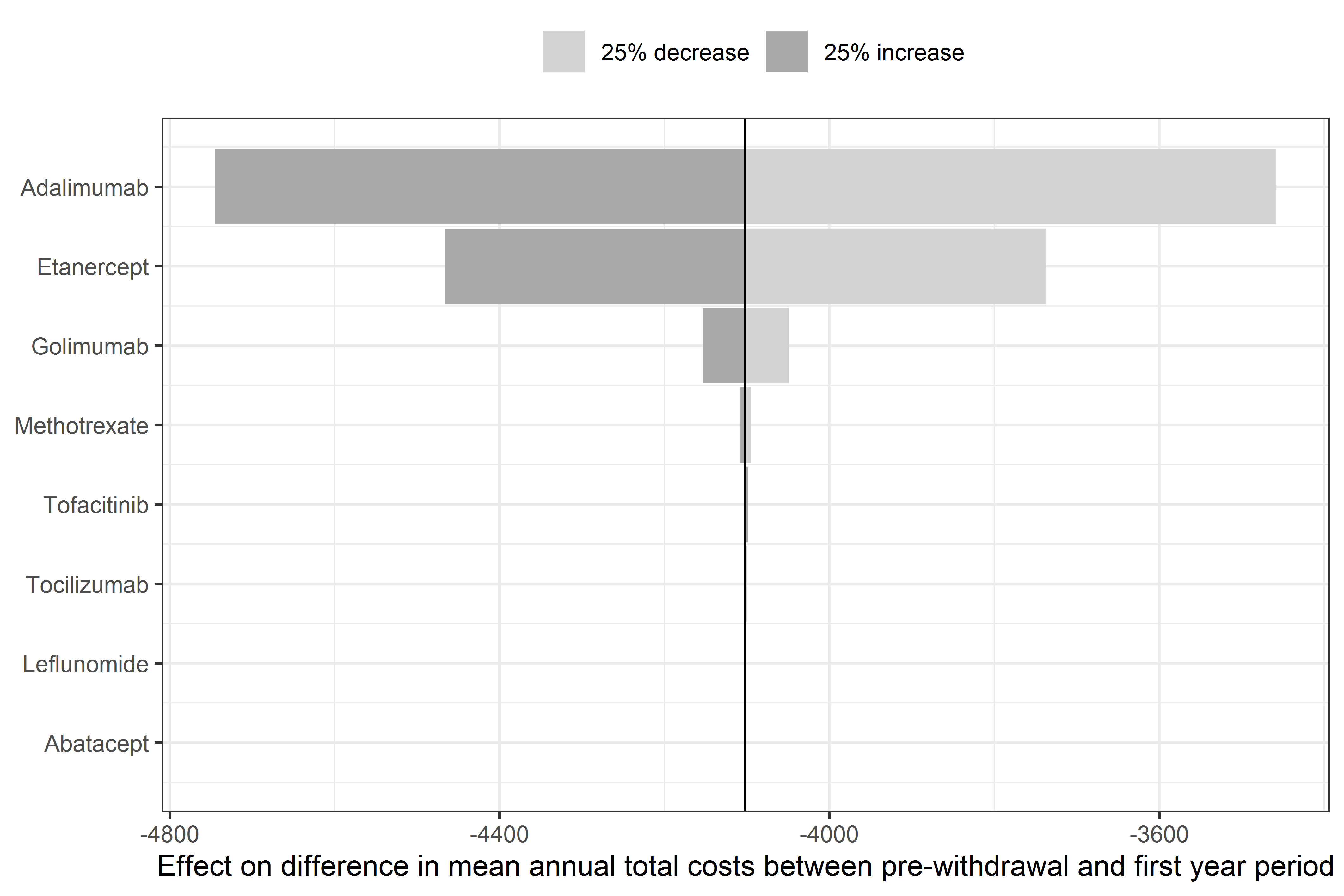


1. **Sensitivity Analysis – Pre-withdrawal vs. Second Year**


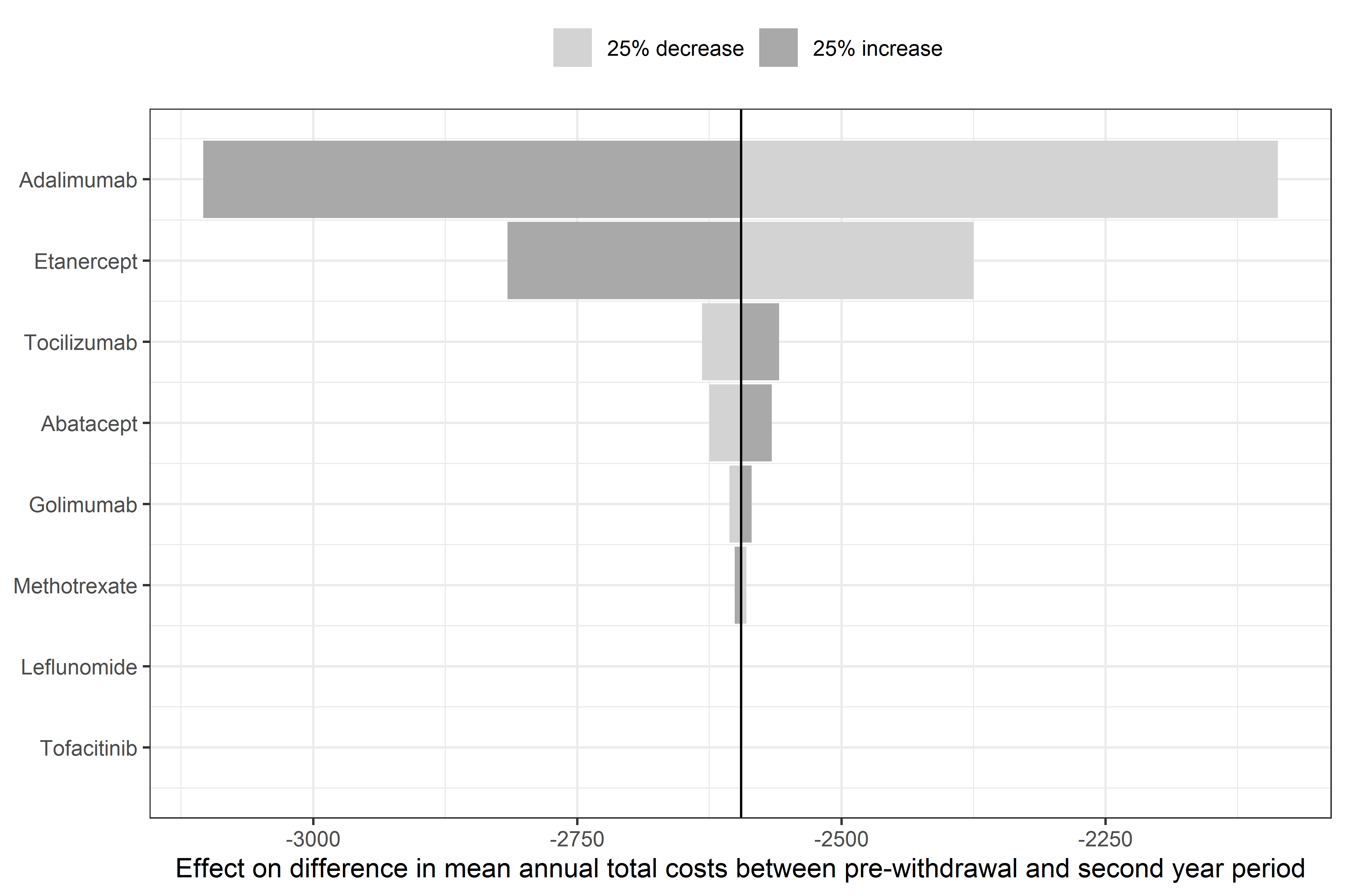


**Supplementary Figure S5 The results of the sensitivity analysis of the DMARD types. A) the effect of varying the cost inputs per DMARD by [-25% (ligth grey), +25% (dark grey)] on the mean annual total cost difference between the period before starting withdrawal and the first year post-withdrawal. B) the effect of varying the cost inputs per DMARD by [-25% (light grey), +25% (dark grey)] on the mean annual total cost difference between the pre-withdrawal period and the second year post-withdrawal.**

## Reference list

1. Zorgproducten [Internet]. Nederlandse Zorg Autoriteit. 2022 [cited 2022 May 20]. Available from: https://zorgproducten.nza.nl/

2. Leona Hakkaart-van Roijen N van der Linden. Kostenhandleiding: Methodologie van kostenonderzoek en  referentieprijzen voor economische evaluaties  in de gezondheidszorg. 2019.

3. Hospital Wilhemina Children’s. Parking Costs [Internet]. 2022 [cited 2022 May 19]. Available from: https://www.hetwkz.nl/nl/parkeren-wkz

4. Zorginstituut-Nederland. Farmacotherapeutisch Kompas [Internet]. 2022. [cited 2022 May 20]. Available from: https://www.farmacotherapeutischkompas.nl/
